# Identification and validation of glomerulotubular crosstalk genes mediating IgA nephropathy by integrated bioinformatics

**DOI:** 10.1186/s12882-022-02779-7

**Published:** 2022-04-13

**Authors:** Yawen Bai, Yajing Li, Yali Xi, Chunjie Ma

**Affiliations:** Traditional Chinese Medicine College, Inner Mongolia Medical University, Jinshan Development District, Hohhot, 010110 People’s Republic of China

**Keywords:** IgA nephropathy, Bioinformatics, Glomerulotubular crosstalk

## Abstract

**Background:**

IgA nephropathy (IgAN), which has been reported as the most prevalent glomerulonephritis globally, is the major contributor to end-stage renal diseases. This bioinformatics study aimed to explore glomerulotubular crosstalk genes and dysregulated pathways relating to the pathogenesis of IgAN.

**Methods:**

The microarray datasets from the Gene Expression Omnibus (GEO) database were searched. Weighted gene co-expression network analysis (WGCNA) and differentially expressed genes (DEGs) of both glomeruli and tubulointerstitium were conducted individually. The co-expression gene modules of glomeruli and tubulointerstitium were compared via gene function enrichment analysis. Subsequently, the crosstalk co-expression network was constructed via the STRING database and key genes were mined from the crosstalk network. Finally, key genes were validated using another GEO dataset (GSE99340) containing RNA-seq data of IgAN and lupus nephritis, and their potential diagnostic values were shown using receiver operating characteristic (ROC) analysis.

**Results:**

Five hundred eighty-three DEGs and eight modules were identified in glomerular samples, while 272 DEGs and four modules were in tubulointerstitial samples. There were 119 overlapping DEGs between the two groups. Among the distinctive modules, four modules in glomeruli and one module in tubulointerstitium were positively associated with IgAN. While four modules in glomeruli and two modules in tubulointerstitium were negatively associated with IgAN. The top ten key genes screened by CytoHubba were ITGAM, ALB, TYROBP, ITGB2, CYBB, HCK, CSF1R, LAPTM5, FN1, and CTSS. Compared with lupus nephritis, there were significant differences in the expression levels of CYBB, CTSS and TYROBP (*P* < 0.05), while other key genes showed no significant difference. Meanwhile, CYBB, CTSS, and TYROBP demonstrated possible diagnostic significance.

**Conclusions:**

The crosstalk genes confirmed in this study may provide novel insight into the pathogenesis of IgAN. Immune-related pathways are associated with both glomerular and tubulointerstitial injuries in IgAN. The glomerulotubular crosstalk might perform a role in the pathogenesis of IgAN.

**Supplementary Information:**

The online version contains supplementary material available at 10.1186/s12882-022-02779-7.

## Background

Immunoglobulin A (IgA) nephropathy (IgAN), which is identified as the most prevalent primary glomerular disease in many countries, is the major contributor to kidney failure. Specifically, end-stage renal disease (ESRD) will develop in 20% to 40% of IgAN patients within 20 years after disease initiation, according to recent research [1, 2]. IgAN is predominantly prevalent among young individuals between the ages of 20 and 40 years and causes a great burden to individuals and societies [2]. Currently, scholars believe that genetic, environmental, and immune factors together determine the occurrence and development of IgAN [3], but the precise pathogenic mechanisms have not been elucidated. Even though IgAN has traditionally been thought of as a mesangial proliferative glomerular disorder, recent research has revealed that tubulointerstitial damage might be more strongly associated with disease progression than glomerulonephritis. Tubular atrophy/interstitial fibrosis has been reported to independently serve as a risk indicator of IgAN progression among patients with this disease. Moreover, tubulointerstitial injury makes glomeruli sensitive to injury and decreases glomerular filtration in chronic kidney diseases [4]. However, the potential genes and signaling pathways through which glomeruli and tubulointerstitium influence each other and promote the occurrence and progression of IgAN are still unclear. Moreover, the treatments for IgAN are based mostly on nonspecific blockers of the renin-angiotensin system (RAS), which are not always effective [5]. The efficacy and safety of immunosuppressants have also long been controversial. Therefore, new therapeutic strategies aiming to interfere with glomerulotubular crosstalk may shed new light on IgAN treatment.

A huge amount of data has been generated and preserved in publicly available databases in recent years as a result of the widespread use of genome transcriptome analysis, such as the Gene Expression Omnibus (GEO) database. The data has been generally applied and used in multiple disease research areas, especially in cancer [6]. Weighted gene co-expression network analysis (WGCNA) is an innovative and potent tool, especially in generating co-expression gene modules from mRNA microarray datasets [7]. The detected gene modules may be utilized for subsequent analyses, including biological functions and key genes identification. Although some bioinformatic studies have been applied to exploring potential molecular mechanisms and therapeutic targets in IgAN [8–10], few studies focus on the shared and different genes of glomeruli and tubulointerstitium of IgAN.

In this study, we aimed to compare similarities and differences between genes of glomeruli and tubulointerstitium, and revealed crosstalk genes underlying the molecular mechanism of IgAN. Firstly, gene expression datasets of glomeruli and tubulointerstitium from IgAN patients as well as corresponding controls were obtained from the GEO database. We then detected the differentially expressed genes (DEGs) of two groups, and the overlapping part was screened. The co-expression networks of glomerular and tubulointerstitial samples were constructed with the help of WGCNA. Then, functional enrichment analysis was performed on the DEGs in each co-expression gene module. Subsequently, the crosstalk network of glomeruli and tubulointerstitium was formed via the String database and key genes were mined from the crosstalk network by CytoHubba in Cytoscape. Lastly, an additional GEO dataset (GSE99340) was utilized to verify the identified key genes.

## Methods

### Ethical compliance

The research was carried out in strict accordance with the *Declaration of Helsinki* (2013). Neither animal experiments nor human clinical trials were conducted as part of our investigation. The raw datasets were available from the GEO database.

### Acquisition, quality control, and processing of data

**Fig. 1:**
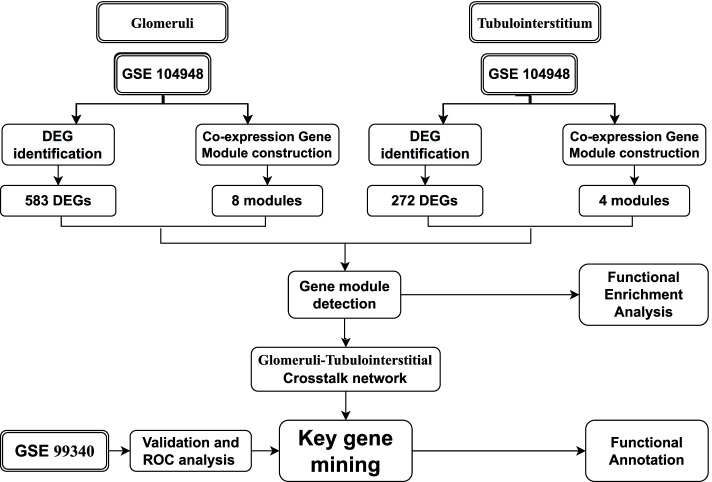
Flow chart of our study

Figure 1 depicts the flowchart of the present research. The GEO database was used to obtain datasets series matrix files comprising GSE104948, GSE104954, and GSE99340. GSE104948 and GSE104954 contained gene expression profiles from glomeruli and tubulointerstitium of IgAN patients and normal controls. GSE99340 containing RNA-seq data of IgAN samples and lupus nephritis (LN) samples were chosen for validation of key genes. Table 1 provides the details of each dataset. Platform documents of GPL22945, GPL24120 [11], GPL19184, and GPL19109 [12] were downloaded to annotate the gene expression. The gene expression matrices were generated with column names as gene symbols and row names as group names for subsequent analysis. Individual datasets underwent quality control by principal component analysis (PCA) using the stats package in R (version 3.6.0) to identify data distribution characteristics.

**Table 1:** Microarray datasets information

Dataset ID	Platform	Sample Details	Sample
GSE104948	GPL22945 GPL24120	Glomeruli from renal biopsy	27 vs21(IgAN vs Control)
GSE104954	GPL22945 GPL24120	Tubulointerstitium from renal biopsy	25 vs 21 (IgAN vs Control)
GSE99340	GPL19184 GPL19109	Glomeruli and tubulointerstitium from renal biopsy	50 vs 60 (IgAN vs LN)

### Screening of differentially expressed genes (DEGs)

The limma package in R (version: 3.5.3) was performed to detect DEGs of glomeruli, tubulointerstitium, and normal tissues. Genes with adjusted *P*-value < 0.05 and |log2(Fold Change) | > 1.5 were considered DEGs.

### Co-expression gene modules constructing

As a bioinformatics approach, WGCNA was utilized to create a scale-free network according to gene expression profiles [13]. All genes in a module are groups of genes with similar patterns of expression [14]. In this research, the WGCNA algorithm was conducted to construct the co-expression modules of glomeruli and tubulointerstitium individually, and then examine the association between the gene modules and disease.

Firstly, outlier samples were filtered using the WGCNA package, and correlation matrices were created afterward. To assess whether the genes exhibited comparable expression patterns, the Pearson correlation coefficient between them was computed and the screening cutoff value was used. Furthermore, the correlation matrices were transformed into a topological overlap matrix (TOM), which could measure the network connection of a gene with all other genes for network gene ratio. To divide genes that have comparable expression patterns into gene modules, we performed the average link hierarchical clustering in accordance with the TOM-based dissimilarity measure using the least size of 20 (tubulointerstitial samples were set to 40) for the genes dendrogram. In addition, we combined modules with a distance of less than 0.6. Finally obtained eight co-expression modules (four modules in tubulointerstitial samples). It should be noted that the grey module is considered to be a set of genes that cannot be assigned to any module. Then, we computed the gene significance (GS) and module membership (MM) and performed Pearson correlation analysis to assess the association between modules and clinical traits (IgAN and control).

### Function annotation analysis of co-expression gene modules

For further insight into the potential mechanisms of co-expression gene modules, DEGs regarding each module (except the grey module) were performed in gene ontology (GO) analysis to reveal their biological function via DAVID (http://ncifcrf.gov). The biological process (BP) of the Go term was focused (The cut-off was adjusted as count > 4, and *p* < 0.05).

### Crosstalk network construction and key genes selection

The crosstalk network of glomeruli and tubulointerstitium was constructed by mapping the hub genes regarding each module. The hub genes were defined as GS > 0.6. Then we used the STRING database (http://string-db.org) to construct the crosstalk network at the protein level. The CytoHubba plugin in Cytoscape software (version3.9.0) was used to screen key genes from the crosstalk module.

### Functional annotation and validation of key genes

To understand the biological functions and signaling pathways of key genes, the genes were uploaded to Metascape (www.metascape.org) [15]. Pathway with *p* ≤ 0.01 and Min Overlap ≥3 was considered statistically significant.

In order to verify key genes, another dataset (GSE99340) containing RNA-seq data of IgAN samples and LN samples was used to confirm whether the identified key genes are specific or not. In this procedure, LN was set as the disease control group, for both IgAN and LN are immunity-related renal diseases, while the specific mechanisms remain different [16]. We performed independent-group t-tests to determine whether there was a substantial difference in gene expression between IgAN and LN (*P* < 0.05). Once this was completed, a receiver operating characteristic (ROC) analysis was carried out to determine the possible diagnostic performance of the genes.

### Statistical analysis

The statistical analyses were carried out utilizing SPSS (version: 19.0) and R (version: 3.5.3). Statistical significance was described as a *p*-value < 0.05.

## Result

### DEGs screening

**Fig. 2:**
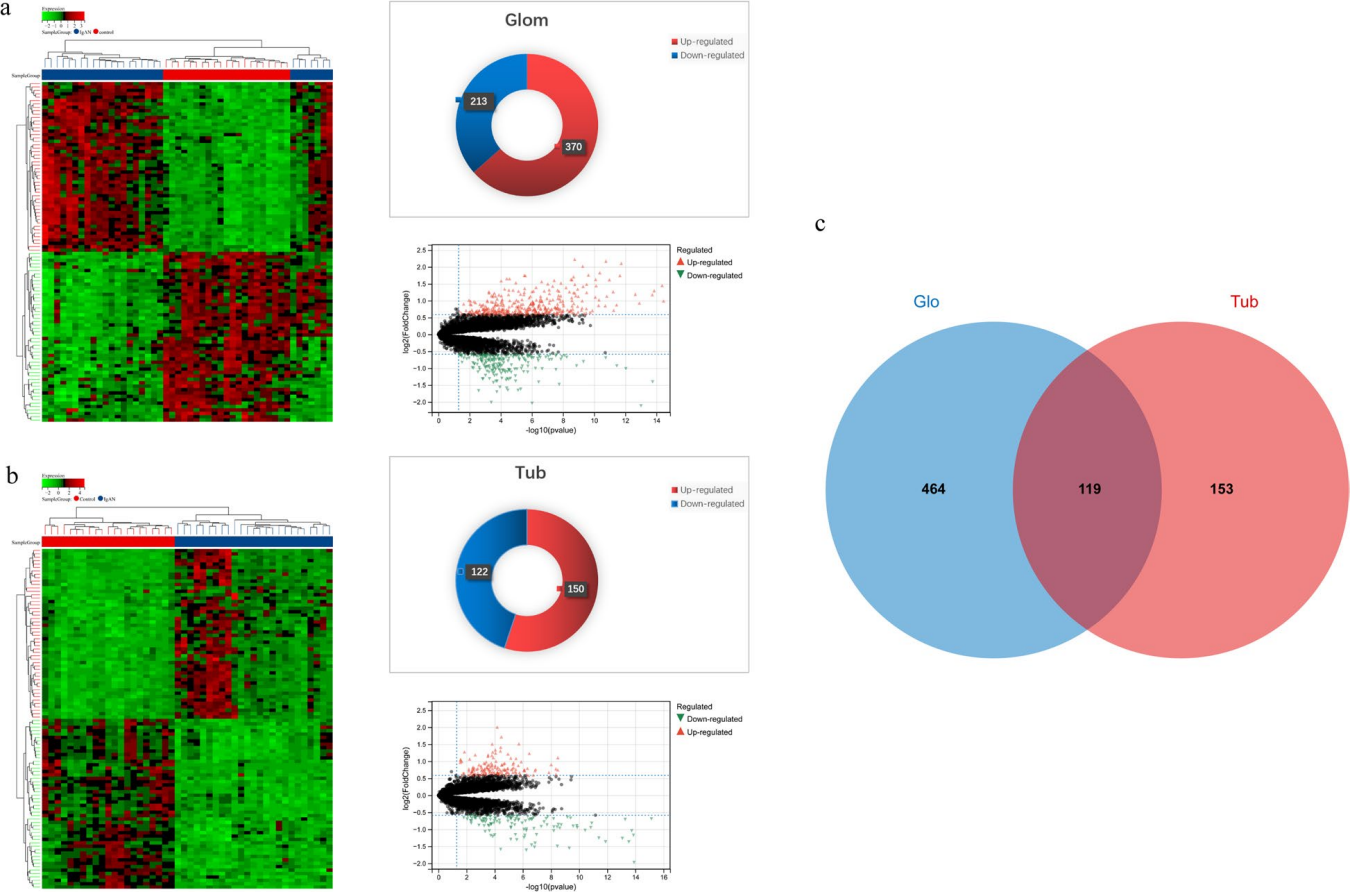
Differential expression genes (DEGs) in glomeruli (Glom) and tubulointerstitium (Tub). **a** The heat map and volcano plot of DEGs in glomeruli (|log2(Fold Change) | > 1.5; adjusted *P* < 0.05). Pie charts represent the numbers of genes found to be upregulated or downregulated. **b** The heat map and volcano plot of DEGs in tubulointerstitium (|log2(Fold Change) | > 1.5; adjusted *P* < 0.05). Pie charts represent the numbers of genes found to be upregulated or downregulated. **c** Venn diagram shows overlapping DEGs in glomeruli and tubulointerstitium

The PCA results showed there were significant differences in sample distributions between the disease group and the control group. In glomerular samples, 583 DEGs were detected, which comprised 213 down-regulated genes and 370 up-regulated genes. Meanwhile, 272 DEGs were identified in tubulointerstitial samples, including 122 down-regulated genes and 150 up-regulated genes (Fig. 2 a, b) (Additional file 1). Among these DEGs, 119 genes were overlapping in both groups (Fig. 2 c).

### Co-expression gene modules constructing

**Fig. 3:**
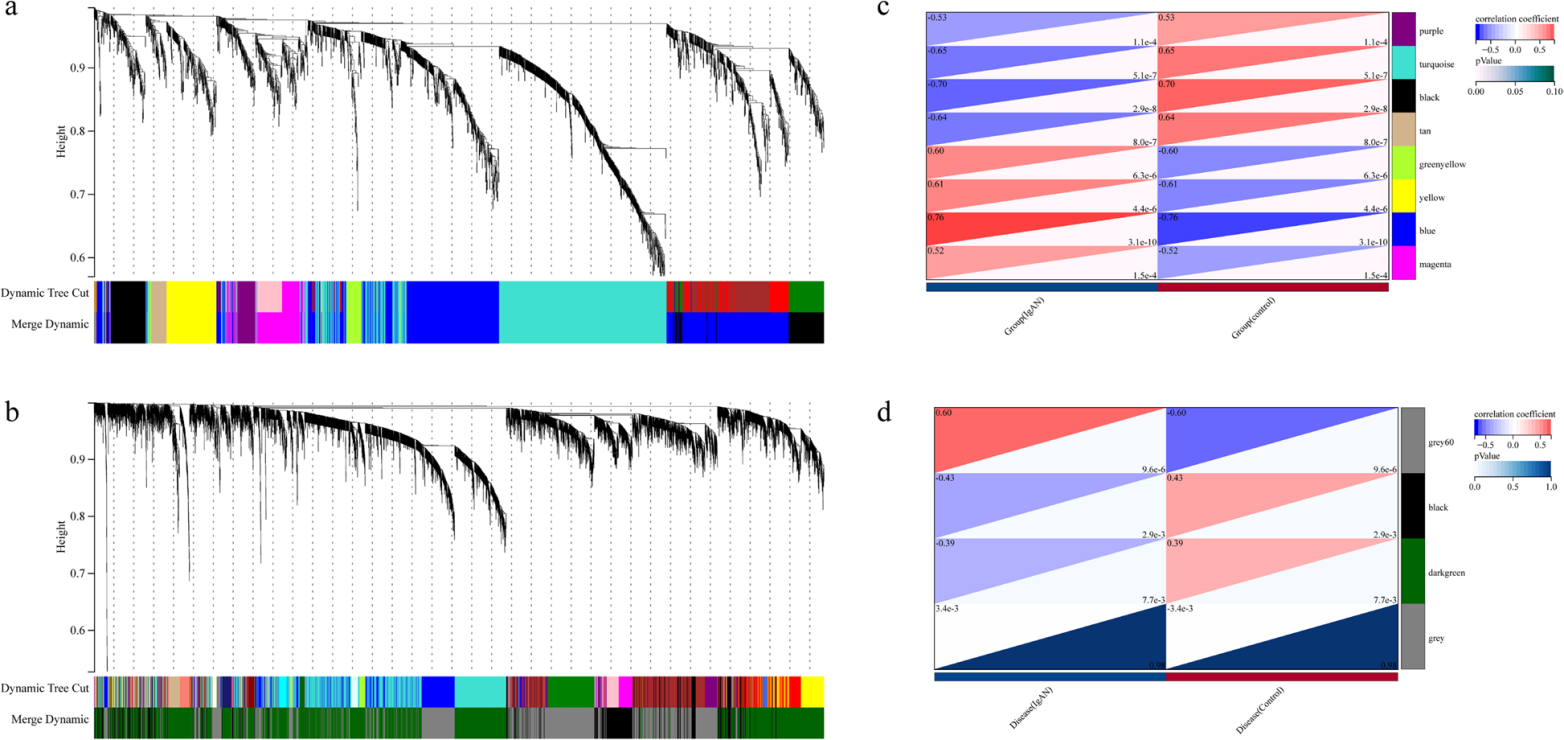
Weighted gene co-expression network analysis. **a** The cluster dendrogram of co-expression genes in glomeruli. **b** The cluster dendrogram of co-expression genes in tubulointerstitium. **c** Module–trait relationships in glomeruli. Each cell contains the corresponding correlation and *p*-value. **d** Module–trait relationships in tubulointerstitium. Each cell contains the corresponding correlation and *p*-value

We detected eight distinctive modules in glomeruli, among them four modules “blue” “yellow” “green-yellow” and “magenta” were positively associated with IgAN, while four modules “purple” “tan” “black” and “turquoise” were negatively associated with IgAN (Fig. 3 a). Similarly, four gene modules in tubulointerstitium were detected (excluded a grey module assigning to no cluster). The “grey60” module was positively associated with IgAN, and two modules “black” and “dark-green” were negatively associated with IgAN (Fig. 3 b). Figure 3 (c, d) depicts heatmaps of the association between each module and a clinical trait (IgAN and normal control).

### Functional enrichment analysis of co-expression gene modules

The DEGs in blue and green-yellow modules that had a positive correlation with IgAN in glomerular samples were found to be enriched in the cellular response to tumor necrosis factor (TNF), type I interferon signaling pathway, and chemokine-mediated signaling pathway. The DEGs in the yellow module were enriched in vasculogenesis. The DEGs in the dark-green module negatively correlated with IgAN were enriched in ion transmembrane transport and drug metabolism (Table 2). In tubulointerstitial samples, the grey60 module positively correlated to IgAN was associated with complement activation and bacterial infection. The negatively correlated module dark-green was enriched in response to cAMP and hormone stimulus, skeletal muscle cell differentiation, and pathways induced by viral infection (Table 3).

**Table 2:** GO-BP enrichment analysis of DEGs in co-expression modules of glomeruli

Modules	The Number of DEGs	Go-BP Terms^a^	***P***-Value
Blue Module	311	GO:0070098:chemokine-mediated signaling pathway	3.38E-09
		GO:0090026:positive regulation of monocyte chemotaxis	5.93E-06
		GO:0071356:cellular response to tumor necrosis factor	2.33E-05
		GO:0070374:positive regulation of ERK1 and ERK2 cascade	2.66E-04
		GO:0002755:MyD88-dependent toll-like receptor signaling pathway	2.52E-04
Yellow Module	28	GO:0000122:negative regulation of transcription from RNA polymerase II promoter	4.39E-06
		GO:0003151:outflow tract morphogenesis	3.74E-05
		GO:0035050:embryonic heart tube development	2.03E-04
		GO:0001570:vasculogenesis	0.003
Greenyellow Module	19	GO:0060337:type I interferon signaling pathway	1.87E-11
		GO:0009615:response to virus	4.54E-08
		GO:0051607:defense response to virus	3.45E-07
		GO:0032728:positive regulation of interferon-beta production	2.95E-04
		GO:0045087:innate immune response	0.007
Magenta Module	5	–	–
Turquoise Module		GO:0035435:phosphate ion transmembrane transport	9.21E-04
		GO:0035725:sodium ion transmembrane transport	0.001
		GO:0006817:phosphate ion transport	0.004
Black Module	25	–	–
Tan Module	1	–	–
Purple Module	0	–	–

^a^The top five terms were displayed according to *p*-value

**Table 3:** GO-BP enrichment analysis of DEGs in co-expression modules of tubulointerstitia

Modules	The Number of DEGs	Go-BP Terms^a^	*P*-Value
Grey60 Module	158	GO:0006958:complement activation	1.55E-06
		GO:0019731:antibacterial humoral response	2.93E-05
		GO:0050829:defense response to Gram-negative bacterium	8.72E-05
		GO:0019882:antigen processing and presentation	8.72E-05
		GO:0033209:tumor necrosis factor-mediated signaling pathway	4.28E-04
Darkgreen Module	114	GO:0051591:response to cAMP	1.11E-05
		GO:0035914:skeletal muscle cell differentiation	2.74E-04
		GO:0032870:cellular response to hormone stimulus	0.003
		GO:0071277:cellular response to calcium ion	0.004
		GO:0042493:response to drug	0.014
Black Module	0	–	–

^a^The top five terms were displayed according to *p*-value

### Crosstalk network construction and key genes selection

**Fig. 4:**
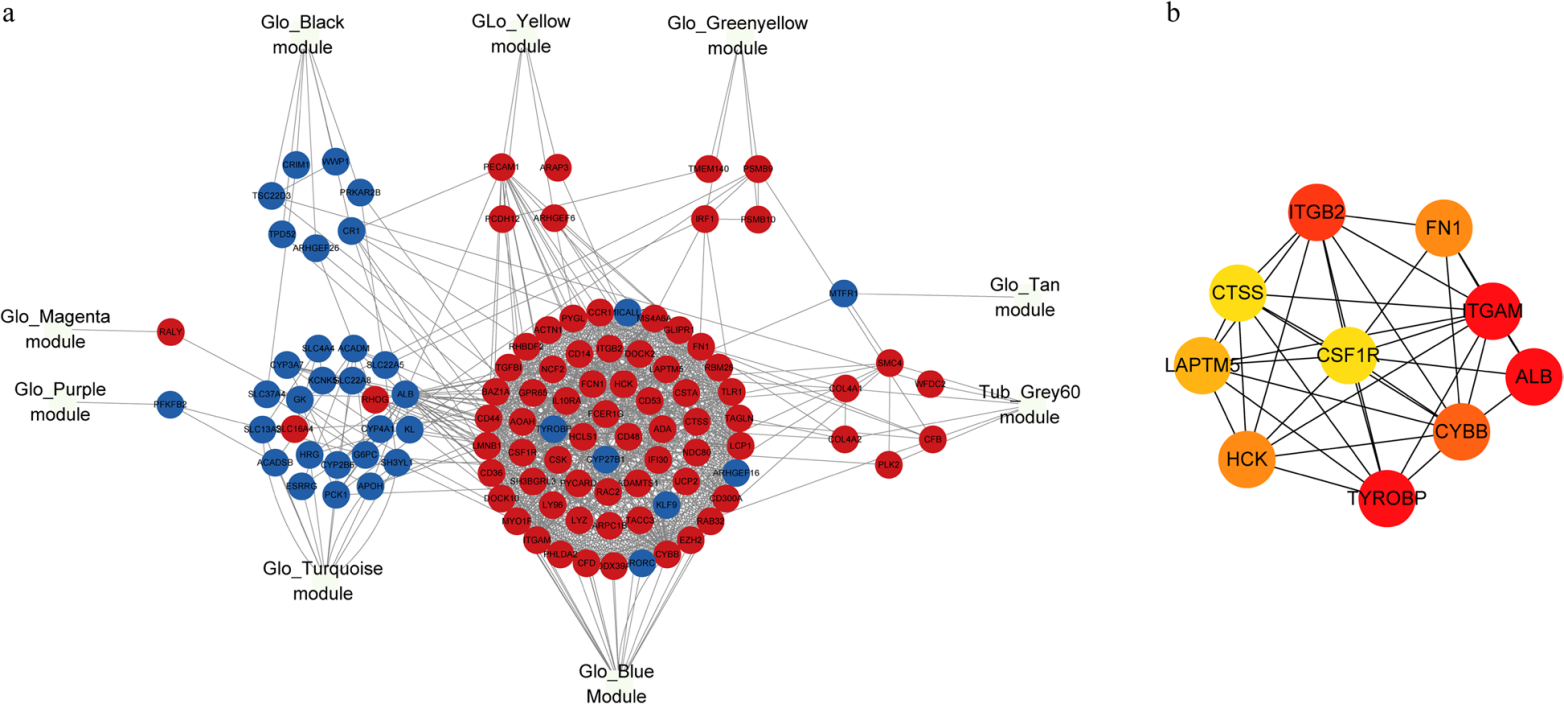
Glomeruli-tubulointerstitium crosstalk network and key genes screened by CytoHubba. **a** Down-regulated genes were in blue while up-regulated genes were in red. **b** Key genes screened by CytoHubba

The crosstalk network of glomeruli and tubulointerstitium was generated by the String database (Fig. 4a). Key genes were screened by the CytoHubba plugin in Cytoscape (Fig. 4b).

### Functional annotation and validation of key genes

**Fig. 5:**
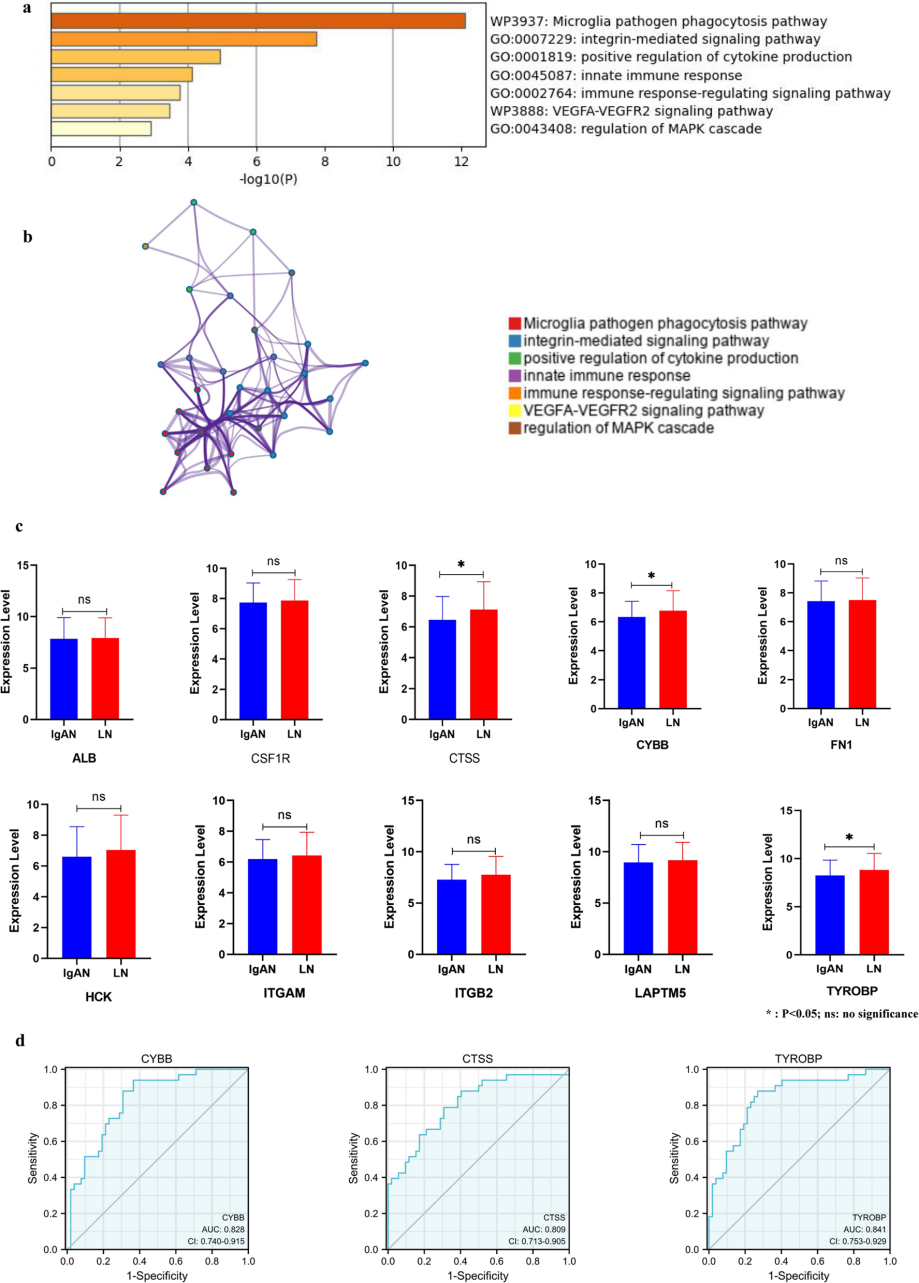
Functional Annotation and Validation of Key Genes. **a** The bar graph displayed the top-level enriched terms for the biological functions and signaling pathways by Metascape analysis. **b** The network diagram of enriched terms. **c** The expression of key genes in datasets GSE99340. **d** ROC curves for specific key genes

Metascape analysis displayed top-level enriched terms as a bar graph for the biological functions and signaling pathways. It identified that the key genes were mainly involved in the integrin-mediated signaling pathways, positive regulation of cytokine production, innate immune response, immune response-regulating signaling pathways, and so on (Fig. 5a). The network diagram was constructed with each enrichment term as a node and the similarity of the node as the edge (Fig. 5b). Among them, the biological processes related to immune response were mainly enriched in positive regulation of myeloid leukocyte mediated immunity, integrin cell surface interactions, fibrin complement receptor 3 signaling pathway. Therefore, we inferred that the immune response might play an important role in IgAN.

GSE99340 was utilized to verify these key genes. Using SPSS (version: 19.0), it was discovered that there were significant differences in the expression levels of CYBB, CTSS and TYROBP between the IgAN group and LN group (*P* < 0.05), while other key genes showed no significant difference (Fig. 5c). The result revealed that the key genes identified in this study were shared partially between the IgAN and LN, while CYBB, CTSS, and TYROBP were specific for IgAN.

Finally, the potential clinical values of CYBB, CTSS, and TYROBP were shown by ROC analysis. All of them demonstrated possible diagnostic values (*P* < 0.05) (Fig. 5d).

## Discussion and conclusion

IgAN, the most common glomerulonephritis worldwide, has a high risk of progression to ESRD [2]. The progression of IgAN is not completely related to glomerular lesions, for example, in some patients with controlled glomerular lesions, the renal function continues to decline, which implies that tubulointerstitial injury may play a role. The Oxford MEST (interstitial fibrosis/tubular atrophy, segmental sclerosis, hypercellularity, endocapillary, and mesangial) histologic score in IgAN suggested that T, S, and M lesions are related to the prognosis of the disease [2, 17]. Although guidelines recommend histological risk factors and clinical features so that disease prediction and therapy selection can be applied properly, specific genes related to glomerular and renal tubular damage are still poorly understood. The mechanisms of crosstalk between glomeruli and tubulointerstitium in the pathogenesis of IgAN are lacking. Therefore, we performed integrated bioinformatics analysis to identify key genes and explore the correlation between glomeruli and tubulointerstitium in the pathogenesis of IgAN.

In the present research, 583 DEGs were detected in glomeruli, comprising 213 down-regulated genes and 370 up-regulated genes. Meanwhile, 272 DEGs were detected in tubulointerstitium, including 150 up-regulated genes and 122 down-regulated genes. Among these DEGs, 119 genes were overlapping in both groups (Fig. 2). The overlapping DEGs suggested that both glomerular and tubulointerstitial lesions may be caused by the same genes and pathways, which could explain that two lesions always occur concurrently in IgAN nephropathy.

Based on WGCNA, eight and four co-expression modules were detected in glomerular and tubulointerstitial samples (Fig. 3), respectively. The positively related modules were “blue” “yellow” “green-yellow” and “magenta” in glomerular samples and “grey60” module in tubulointerstitial samples. While the negatively related modules were “black”, “tan”, “turquoise” and “purple” modules in glomerular samples and “dark-green” and “black” modules in tubulointerstitial samples.

Through gene functional enrichment analysis of the positively related gene modules, we found that both glomeruli and tubulointerstitium were involved in the adaptive and innate immune systems. Since bacterial or viral infection may trigger the occurrence of IgAN and recurrent infections may worsen the disease. Previous studies have shown that streptococcus may aggravate inflammatory damage in IgAN via the chemotaxis of Th22 cells [3]. The activation of Toll-like receptors (TLRs) might promote the production of IgA and elevate IgA glycosylation. Moreover, TLR 9 and TLR 4 were correlated with IgAN severity [18]. The yellow module in glomeruli was related to vasculogenesis, which suggested that vascular endothelial growth factor (VEGF) and other related inflammatory factors may lead to inflammation and proliferation of the mesangial cells and further cause glomerulosclerosis [19]. Plate-derived growth factor (PDGF) not only directly stimulates the proliferation of mesangial cells but also can lead to renal fibrosis [20]. The grey60 module in tubulointerstitial samples positively correlated with IgAN was predominately enriched in the complement activation pathway. It has been confirmed that in human and rodent experiments, IgA (mainly poly IgA involved) can activate complement alternative pathway (AP) [21]. The modules negatively related to IgAN indicated that the biological process of ion transmembrane transport has a protective effect on IgAN.

Concerning the pathogenesis of IgAN, the “multi-hit” hypothesis, including production of galactose-deficient IgA1 (Hit 1), anti-glycan response (Hit 2), formation of IgA1-containing immune complexes (Hit 3), and glomerular deposition (Hit 4), has been widely supported by many studies [22]. Tubulointerstitial damage in IgAN is considered to be a secondary event of glomerular injury developing later in IgAN. However, a recent single-cell RNA sequencing (scRNA-seq) study revealed that proximal tubular cells are injured already at the early stage of IgAN [23]. This may be partially mediated by albuminuria and glomerulotubular cross-talk. Meanwhile, the tubulointerstitial injury may also lead to increased glomerular damage. Tubulointerstitial fibrosis can activate the macula densa and result in abnormal tubuloglomerular feedback, with continuing arteriolar vasodilation, and consequently result in glomerulosclerosis [24]. Therefore, we propose that this adverse cross-talk of glomeruli and tubulointerstitium will enhance IgAN progression in all stages.

In our study, the key genes screened from the crosstalk network were mainly associated with adaptive and innate immunity, such as ITGAM, ITGB2, TYROBP, CSF1R, HCK, and LAPTM5. As mentioned above, the “first hit” is that individuals with a genetic susceptibility develop abnormal immune responses to common and environmental antigens, which leads to an increase of galactose-deficient IgA1 in circulation. A growing body of evidence suggests that abnormality of intestinal mucosal immunity plays an important role in IgAN [25]. Integrin alpha M (ITGAM) encodes the integrin alpha M chain (CD11b) and belongs to the integrin family. CD11 is an important leukocyte differentiation antigen, which is widely expressed in a variety of immune cell subsets, such as dendritic cells, neutrophils, NK cells, and B cells. CD11 integrin participates in innate immunity, adaptive immunity, and inflammatory response, and plays an important role in regulating immune tolerance [26, 27]. ITGAM is proven to participate in the modulation of intestinal IgA-producing plasma cells in mice, which indicates the function of the intestinal immune in the pathogenic mechanism of IgAN [27]. Integrin subunit beta 2 (ITGB2) also belongs to the integrin family and is implicated in binding between endothelial cells and inflammatory cells, inflammatory cells chemotaxis [28]. According to a previous study, there is a negative correlation between ITGB2 and eGFR in patients with chronic kidney disease (CKD). However, the mechanisms of ITGB2 mediating glomerular and tubulointerstitial injuries are not clear, which needs further study. Transmembrane immune signaling adaptor (TYROBP) encodes a transmembrane signaling polypeptide that encompasses an immunoreceptor tyrosine-based activating motif in its cytoplasmic domain. TYROBP binds non-covalently to NK cell activity receptors and activates signal transduction. It has been previously reported that TYROBP is highly correlated with proteinuria in systemic lupus erythematosus (SLE) [29], but its role in IgAN is unclear. Cytochrome b-245 beta chain (CYBB) has been postulated as a major constituent of the phagocyte microbicidal oxidase system [30]. Fibronectin 1 (FN1) is a well-known protein that has great binding activity and is also the primary constituent of the extracellular matrix. The aggregation and chemotaxis of FN1 and collagen serve as critical building blocks for the proliferation of endothelial cells, mesangial cells, and fibroblasts. The expression of FN1 in the glomeruli indicates the presence of active mesangial cell growth and the progression of the lesion [31]. Cathepsin S (CTSS) participates in antigen presentation and cytokine secretion. It has been demonstrated that CTSS inhibits apoptosis and promotes cell proliferation through PI3K/Akt or MAPK pathway [32]. A recent study on proteomics of the tubulointerstitium in IgAN shows that the abundance of cathepsin G (CTSG) was related to disease progression [33]. Both CTSG and CTSS belong to the cathepsins super-family, which are lysosomal enzymes that participate in important physiological processes, such as tissue remodeling, senescence and adaptive and innate immunity. Hemopoietic cell kinase (HCK) plays an important part in regulating innate immune response, phagocytosis, cell survival and proliferation, cell adhesion, and migration [34]. HCK can activate TGF-*β*–mediated pro-fibrotic pathway, as well as other proliferation contributing factors, which are implicated in renal tubular cell damage and fibrosis and even the modulation of the immune system [35]. It has been demonstrated that lysosomal protein transmembrane 5 (LAPTM5) performs an instrumental function in the lysosomal disintegration of B cell and T cell antigen receptors (BCR/TCR) by transporting endosomes to lysosomes.

Based on the analysis presented above, we hypothesize that inflammatory mediators (cytokines and chemokines) released by ligand-receptor interactions between glomeruli and tubulointerstitium, together with albuminuria, may participate in glomerulotubular crosstalk.

In our study, another dataset was used to verify the key genes, and LN was served as the control group. The result revealed that key genes were shared partially between IgAN and LN, while CYBB, CTSS and TYROBP were specific for IgAN. Both IgAN and LN are immunity-related renal diseases and share many similarities. For example, both have proteinuria and hematuria as common clinical manifestations, and renal IgA deposition as pathological manifestations. Not only that, co-occurrences of IgAN and LN were found in some patients [36]. The results of our study are basically consistent with the previous studies [16, 37].

As mentioned above, some of the key genes have been shown to perform a vital part in the pathogenic process of IgAN, such as ITGAM [38], ALB [39], FN1 [40] and CTSS [41]. However, there is little known about CYBB, TYROBP, ITGB2, CSF1R, HCK, and LAPTM5, which need further research to reveal their mechanisms.

The limitation of this study. Because of the scarcity of clinical data in the GEO database, it is hard for us to link gene modules to specific clinical characteristics. In addition, the data of our study were obtained by bioinformatic analysis of microarray datasets; consequently, more in vivo and in vitro tests are required to validate the findings.

## Supplementary Information

**Additional file 1.**

## Data Availability

These data were derived from the following resources available in the public domain: Gene Expression Omnibus (GEO) database http://www.ncbi.nlm.nih.gov/geo/.

## Abbreviations

IgAN: IgA nephropathy
LN: Lupus nephritis
DEG: Differentially expressed genes
WGCNA: Weighted gene co-expression network analysis
NC: Normal control
TLRs: Toll-like receptors
LAPTM5: Lysosomal protein transmembrane 5
PDGF: Plate-derived growth factor
VEGF: Vascular endothelial growth factor
ITGAM: Integrin alpha M
ITGB2: Integrin subunit beta 2
CKD: Chronic kidney disease
TYROBP: Transmembrane immune signaling adaptor
SLE: Systemic lupus erythematosus
CYBB: Cytochrome b-245 beta chain
FN1: Fibronectin 1
CTSS: Cathepsin S
HCK: Hemopoietic Cell Kinase
